# Classifying Different Types of Music Performance Anxiety

**DOI:** 10.3389/fpsyg.2021.538535

**Published:** 2021-04-23

**Authors:** Claudia Spahn, Franziska Krampe, Manfred Nusseck

**Affiliations:** ^1^Freiburg Institute for Musicians’ Medicine, University of Music Freiburg, Medical Faculty of the Albert-Ludwigs-University Freiburg, Freiburg Center for Research and Teaching in Music, Freiburg, Germany; ^2^Childrens Hospitals Schwabing and Harlaching Munich, Hospital Rechts der Isar, Technical University Munich, München, Germany

**Keywords:** performance science, music performance anxiety, self-efficacy, coping, performance quality

## Abstract

Music performance anxiety (MPA) is a commonly present topic among musicians. Most studies on MPA investigated effects of a more general occurrence of MPA on performances. Less is known about individual variations of MPA within a performance, more specifically at the times before, during, and after the performance. This study used a questionnaire to investigate these performance times in order to find out if there occur different types in the variation of the perceived MPA across the performance. The study was performed with 532 musicians; 27% of them being professional orchestra musicians, 45% non-professional orchestra musicians, and 28% non-professional choir singers. The musicians were asked to fill in the Performance-specific Questionnaire for Musicians (PQM) immediately after a performance. The questionnaire contains three scales regarding symptoms of MPA, functional coping with MPA and performance-related self-efficacy. A cluster analysis was performed on the PQM scales to identify systematic variations. Findings indicate that there are three different types of MPA in the sample studied. Type 1 describes musicians who have few symptoms of MPA throughout the performance, show functional coping with MPA, and have a stable and well-developed self-efficacy. Type 2 describes musicians who begin their performance with rather high symptoms of MPA but can positively reduce these by the end of the performance and show high values in self-efficacy and in functional coping. Type 3 contains musicians who begin their performance with some symptoms of MPA, which increase to the end of the performance. The values of self-efficacy and functional coping in this type are rather low. Of the total sample, half of the musicians were assigned to Type 1 and approximately a quarter each to Type 2 (27%) and Type 3 (23%). In accordance with the literature, the results confirm the importance of self-efficacy and functional coping for a positive performance experience.

## Introduction

Music performance anxiety (MPA) has the potential to be both facilitating and debilitating to performance outcomes (Osborne and Franklin, 2002). Performers encounter individual forms of MPA related to personal experiences and attitudes. It has been shown that characteristic trait anxiety is strongly associated with MPA (Kenny et al., 2004). The level of MPA embodies combinations of affective, cognitive, psychological, and behavioral symptoms (Kenny, 2011).

Wolfe (1989) performed a study with 162 musicians on the facilitating and debilitating effects of MPA on music performance quality. The study showed that adaptive anxiety could enhance the performance, and musicians who engaged in anxiety-coping behavior tended to be those who experience MPA as facilitative. Maladaptive or debilitating anxiety on the other hand is associated with psychological distress, perceived pressure, and weak confidence (Kenny, 2011). It was shown that debilitating MPA could have negative effects on performance quality (Simoens et al., 2015). High levels of MPA could also lead to chronically debilitating impacts on future performance experiences (Kenny and Osborne, 2006).

Research on MPA also investigated associated factors such as gender, age, and performance context to identify possible predictors of MPA. Where it has been shown that females were significantly more likely to experience MPA than males, these findings vary for different age and performance contexts (Kenny et al., 2014). Similar variations in prevalence of MPA have been found in different age groups (Kenny and Osborne, 2006; Nusseck et al., 2015). However, Wolfe (1989) found that age and gender appeared to have no effect on the manifestation of MPA. Kenny (2011) showed that the level of MPA is independent of years of training, practice, and musical accomplishment. Studies showed that university music students experienced MPA at a similar level to professional musicians (Creech et al., 2009; Spahn, 2015).

Regarding the performance context, it has been shown that solo performances trigger higher levels of MPA than performances in groups (Cox and Kenardy, 1993; Papageorgi et al., 2011). Perceived pressure and stress considering the concerns of the performance success can also increase MPA to a debilitating level (Kenny, 2011). This has also been found to be independent of musical training and level of preparation. For example, an experienced and well-prepared pianist could experience high MPA due to the degree of importance of the performance (Yoshie et al., 2009). Furthermore, Wolfe (1989) found that string players exhibited higher levels of MPA compared to other instrumentalists.

One of the most positive influencing factors on the characteristics of MPA is self-efficacy. Self-efficacy is defined by Bandura (1997) as the belief in one’s own capabilities to organize and execute the courses of action required to produce a given attainment. As a theoretical framework for the predictive power of self-efficacy over MPA, Bandura (1993) asserted that “perceived efficacy to exercise control over potentially threatening events plays a central role in anxiety arousal.”

Music self-efficacy was positively correlated with self-regulatory activities (e.g., concentration, goal setting, and planning while practicing), and the active use of strategies for coping with performance anxiety (McCormick and McPherson, 2003; McPherson and McCormick, 2006; Hewitt, 2015; Miksza, 2015; Bugos et al., 2016). McPherson and McCormick (2006) demonstrated the importance of self-efficacy in predicting young musicians’ performance results. Self-efficacy was found to be the most important predictor of achievement in musical examinations. The authors emphasize that self-efficacy is especially important in a discipline like music that involves high levels of self-regulation and mental discipline to achieve success (McCormick and McPherson, 2003; McPherson and McCormick, 2006). Also, in a recent study by González et al. (2017) with a sample of 270 Spanish musicians, self-efficacy was a positive predictor of performance boost, which is understood as a positive state of extra alertness when performing. The positive relationship found between self-efficacy and performance boost showed similar indices for females, males, students, teachers, and performers in their study sample.

It seems important that MPA within a musical performance context has to be looked at as a process with an explicit time dimension of pre-, during-, and post-performance (Papageorgi et al., 2007). This could mean that self-efficacy also comes into play before, during, and after a musical performance and is decisive for how successful musicians develop the self-assurance needed to approach and manage the challenge of a performance (McPherson and McCormick, 2006). The situation before the performance has a crucial influence on the direction in which MPA develops and on which performance achievement results. Kaleńska-Rodzaj (2018) conducted a study with 94 music school students each of them performing a piece once at an open school audition. With a low MPA before the performance, the personal satisfaction of the students as well as the quality assessment of the students and teachers was significantly better than with a high MPA before the performance.

In a recent study, Osborne and McPherson (2018) investigated the correlation between the attitude before the performance and the success of the performance with 36 Bachelor of Music students. According to Lazarus’ cognitive-motivational-relational theory, it was assumed that emotions may wield powerful consequences depending on whether the performance is interpreted as a threat – with high importance at the primary appraisal and low coping prospects at the secondary appraisal – or as a challenge – with high importance and high coping prospects. It was confirmed in their study that students who viewed the performance as a challenge reported significantly less cognitive anxiety and higher self-confidence.

Most research on MPA has focused on the processes prior and during the musical performance. However, even the perceptions of the musician after the performance will produce long-term effects on performance success in the future. In the area of sports, studies showed that systematic self-reflection of a past performance and the focus of good performances can increase the intrinsic motivation and self-esteem that lead to a positive thinking of the outcome of future performances (Van Lier et al., 2015; Chow and Luzzeri, 2019). For musicians, if the musician perceived the performance as positive, he or she will most likely experience an increase in self-efficacy, confidence, and self-esteem, creating positive preconditions for success on analogous performances in the future. If the perceptions after the performance are negative, this can lead to a negative self-concept, low self-esteem, and low self-efficacy, possibly resulting in intensifying MPA in subsequent performances. Papageorgi et al. (2007) propose a theoretical framework that portrays MPA within a musical performance context as a process that has an explicit time dimension (pre-, during-, and post-performance). The model illustrates the likely processes that occur once a performer agrees to participate in a particular performance and explains how these might give rise to either maladaptive or adaptive forms of performance anxiety. Decisive factors are strategies coping with MPA, which have a positive effect on the impairing symptoms of MPA. The strategies that musicians use to cope with MPA may be important in how successful they are at controlling physiological arousal and alleviating the potential maladaptive effects of MPA. Research has indicated that musicians experiencing adaptive MPA use a combination of coping strategies focusing on maintaining a positive attitude to the performance, concentrating on communication with the audience and enjoyment of the music (Papageorgi et al., 2007).

Studies on MPA also mainly focused on the effect of generally self-perceived degrees of MPA on performances. So far, there is no study considering possible changes in the perceived MPA over the performance. As Kaleńska-Rodzaj (2018) showed that the level of MPA prior to the performance is a certain predictor for the performance rating, it is unclear how the musician perceived MPA during or even after the performance. The aim of our research was to investigate how aspects of MPA may change within the times before, during, and after a performance.

The questionnaire for assessing self-reported MPA (PQM, Spahn et al., 2016) enables the investigation of MPA-related aspects considering a just finished performance and refers retrospectively to the times before, during, and after the performance. In particular, with three scales it addresses aspects of specific symptoms of MPA, the functional coping with MPA, and the self-efficacy. The PQM provides the possibility of identifying different types of MPA according to the variations in the scales over the times of the performance. The questionnaire was used primarily in this study.

Furthermore, the distribution of certain variables such as age and gender of the musicians within the different types of MPA were analyzed. It was assumed according to Wolfe (1989) that there were no differences regarding these demographic variables between the types. However, there are possible differences in the manifestation of MPA between professional and non-professional musicians as well as singers, especially regarding the personal importance and the difficulty of the performance. Differences in the distribution of performer demographics within the types of MPA would confirm these suppositions. Regarding the impact of performing a solo part on the occurrence of MPA (Cox and Kenardy, 1993; Papageorgi et al., 2011), a certain effect of a solo performance across the types of MPA was to be expected. A detailed analysis was also performed on distribution differences across musical instruments. The results of this explorative study provide insights on how musicians experience a performance regarding aspects of MPA and how special characteristic factors show an association with certain types of MPA.

## Materials and Methods

### Participants

A total of 532 musicians participated in the study and filled in the questionnaire. According to the musical level of the orchestra, the musicians were classified into three musical subgroups of professional and non-professional instrumentalists and choir singers. The sample consisted of 26% professional orchestra musicians working at radio symphony orchestras and philharmonic orchestras, 46% non-professional orchestra musicians from student orchestras and semiprofessional orchestras, and 28% amateur choir singers from semiprofessional choruses.

Table 1 shows the demographic data of the total sample and the musical subgroups. The age of the professional orchestra musicians was significantly higher than the age of the non-professional orchestra musicians and the amateur choir singers [*F*(2,516) = 85.07, *p* < 0.001], with the latter two being mostly students. There were more females among the choir singers than among the orchestra musicians. The distribution of the instruments in the orchestra subgroups is also listed in Table 1. They provide a rather representative distribution for classical orchestras similar with other studies (e.g., Kenny et al., 2014). The musicians were asked if they played a solo part during the performance. Significantly more musicians among the professional orchestra musicians had played a solo part during the performance compared to the non-professional orchestra musicians and the amateur choir singers [*χ*^2^(524,2) = 56.99, *p* < 0.001].

**Table 1:** Data of the total sample and the subgroups.

	Professional orchestra musicians (27%; *n* = 141)	Non-professional orchestra musicians (45%; *n* = 241)	Amateur choir singers (28%; *n* = 150)	Total sample (100%; *n* = 532)
Age (in years, Mean/SD)	43.5 (12.9)	26.3 (10.8)	29.9 (14.4)	31.8 (14.3)
Gender female (%)	41.3	55.0	76.0	57.3
**Instruments (%)**				
Strings	75.0	58.6		
Woodwind	13.3	19.8		
Brass	6.7	15.9		
Percussion	5.0	3.9		
Others	0	1.8		
Solo part (%)	42.0	33.2	4.7	

### Measures

Self-reported MPA was measured using the PQM (Spahn et al., 2016). This questionnaire originated in a German version [“Fragebogen zum Auftritt für MusikerInnen” (FZAM)] and has been revised and validated to the fourth version (Biwer, 2015). The example items and the name of the German scales were translated into English for this article.

The questionnaire focuses on different aspects of MPA and refers to a just finished performance. It is required to be completed immediately following the performance. The participants answer questions regarding MPA retrospectively considering the times before and during the performance and the moment when filling in the questionnaire after the performance. The reliability of the pre-performance variables has been validated by Biwer (2015). A state anxiety questionnaire was used directly before the performance and the results showed that the state anxiety correlated significantly with the variables of the PQM before the performance but not with the variables of the PQM after the performance. The retrospective statements regarding the time before the performance can therefore be considered reliable.

The questionnaire contains a total of 42 items. The first 32 items address the three main scales of the PQM: (1) symptoms of MPA (12 items; Cronbach’s alpha = 0.77), referring to the occurrence of MPA specific implications (“I could sense signs of agitation in my body”), (2) functional coping (nine items; Cronbach’s alpha = 0.74), regarding positive activities in handling with MPA (“I managed to control my agitation and stay calm”), and (3) self-efficacy (11 items; Cronbach’s alpha = 0.73), considering one’s own confidence of performing (“I was looking forward to going on stage and showing what I could do”).The answers were given on a five-point Likert scale (1 – “strongly agree” to 5 – “strongly disagree”). All three scales can be assigned to the three performance times of pre-performance, during performance, and after performance. Higher scores in the scale symptoms of MPA indicate higher levels of debilitating MPA. Higher values in the scales functional coping and self-efficacy indicate better coping and higher self-efficacy.

A fourth scale is for self-assessment of musical quality of the performance (seven items; Cronbach’s alpha = 0.88). The items have a prefix of “When considering the musical quality of my performance, I rate…,” followed by seven music-related aspects such as “the dynamic shaping” or “the musical expression.” Items were answered using a six-point scale ranging from 1 = “very poor” to 6 = “excellent.” The scale values were calculated as the mean of the seven items.

The participants were also asked to evaluate how important and difficult the performance was to them with three items. The first question was to state the personal importance of the performance on a four-point scale (“Doing well in this concert is personally 1 – not important, 2 – not so important, 3 – important, and 4 – very important for me”). In the second question, the participants had to rate the compared performance difficulty on a four-point scale (“Compared to other performances this performance was 1 – easy, 2 – not so easy, 3 – rather difficult, and 4 – difficult for me”). The general difficulty of the concert itself was asked in the third question with a four-point answer scale (“The difficulty of this concert was, 1 – too low, 2 – low, 3 – just right, 4 – high, and 5 – too high for me”).

### Procedure

At a total of 15 different concerts, the musicians filled in the questionnaire of this study immediately after the performance. The concerts were public concerts with audiences between 300 and 1,500 persons. The chosen concerts were regular concerts of the respective music group in their standard performance schedule. The musicians were given the questionnaire before entering the off-stage facilities and were asked to complete it before speaking with other persons or musicians about the concert.

This study was granted ethical approval by the Ethics Committee of the University Clinic Freiburg. The questionnaire was completely anonymous, and participation was voluntary with no payment given in exchange for participation.

### Data Analyses

The data analyses were performed using SPSS (Version 26, Armonk, NY: IBM Corp.). Descriptive statistics were calculated for each variable. A hierarchical cluster analysis (Method: single-linkage between groups; Squared Euclidean Distance) was performed on all performance scales of the PQM. The cluster solution was used to perform a k-mean cluster analysis with the same scales. To provide the percentage of explained variance, a discriminate analysis was calculated on the k-mean clusters. Chi-square (*χ*^2^) tests were performed to assess distribution differences of non-parametric variables within the clusters. Parametric comparisons of the PQM scales or the questions on experienced importance and difficulty between clusters have been calculated with MANOVAs. When significant, *post hoc* analyses with Tukey HSD correction were performed. The level of statistical significance was set at 0.05.

## Results

### Cluster Analysis With the PQM Scales

With all scales of the PQM, a hierarchical cluster analysis was performed. The analysis yielded a solution with three clusters. The cluster centers were calculated with a k-mean cluster analysis and the ANOVA showed highly significant differences between the groups in all scales. The discriminant analysis showed 64.5% explained variance. Due to incomplete responses, the data of 10 subjects were not included in the cluster analysis.

The constellations of the PQM scales were clearly different across the clusters and presented dissimilar progressions over the performance time. Table 2 shows the mean values with SD for the three scales of the PQM before, during, and after the performance for each cluster. For the following description of the clusters, values greater than four in the scales functional coping with MPA and self-efficacy were considered as high values, and in the symptoms of the MPA scale values below two were considered as low values.

**Table 2:** Mean values for the three scales of the PQM before, during, and after the performance by clusters.

Variables	Cluster 1 (*n* = 259)	Cluster 2 (*n* = 142)	Cluster 3 (*n* = 121)
**Pre-performance**			
Functional coping	4.58 (0.49)	3.98 (0.63)	3.80 (0.73)
Symptoms of MPA	1.51 (0.46)	2.80 (0.79)	2.11 (0.85)
Self-efficacy	4.16 (0.59)	3.91 (0.51)	3.05 (0.65)
**During performance**			
Functional Coping	4.67 (0.46)	3.99 (0.55)	3.81 (0.75)
Symptoms of MPA	1.38 (0.42)	2.53 (0.69)	1.99 (0.77)
Self-efficacy	4.48 (0.45)	4.06 (0.49)	3.33 (0.57)
**Post-performance**			
Functional Coping	4.64 (0.42)	4.44 (0.41)	3.40 (0.66)
Symptoms of MPA	1.29 (0.41)	1.68 (0.69)	2.37 (0.78)
Self-efficacy	4.44 (0.57)	4.12 (0.57)	3.12 (0.81)

In brackets: SD of the mean.

### Cluster 1

**Figure 1:**
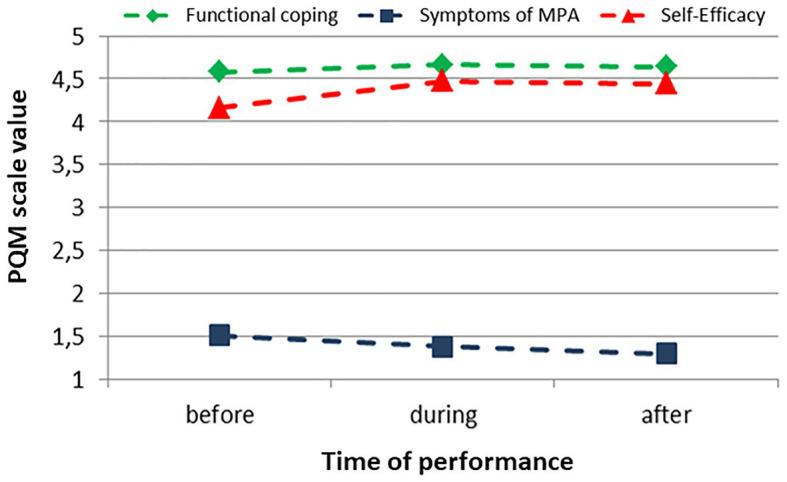
Cluster 1, progression of the values of the Performance-specific Questionnaire for Musicians (PQM) scales before, during, and after the performance (*n* = 259).

The musicians of Cluster 1 had few symptoms of MPA prior to the performance, a very high value in the functional coping with MPA, and at the same time also a very highly pronounced self-efficacy. They began with an optimal constellation in the performance and could also maintain this during the performance. Following the performance, symptoms of MPA were at a very low level, and the participants assessed their performance as positive and felt strong for the next performance. 49.6% (*n* = 259) of the participants were assigned to cluster 1 (Figure 1).

### Cluster 2

**Figure 2:**
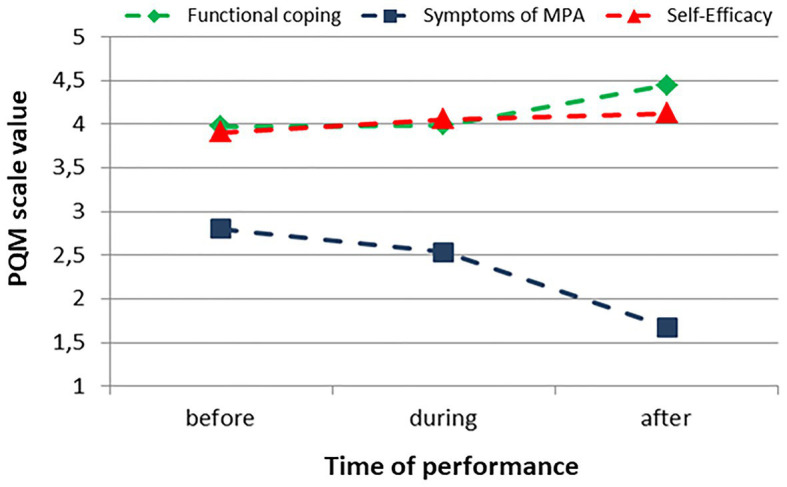
Cluster 2, progression of the values of the PQM scales before, during, and after the performance (*n* = 142).

Participants of Cluster 2 showed a mean value for symptoms of MPA of 2.80 prior to the performance, which is almost twice as high compared to the participants from Cluster 1. Cluster 2 also showed high values for functional coping with MPA and for self-efficacy. With the participants in Cluster 2 the symptoms of MPA during the performance decreased and after the performance they were at a low level. Functional coping and self-efficacy remained stable during the performance and after the performance the functional coping reached the highest value. 27.2% (n = 142) of the participants were assigned to Cluster 2 (Figure 2).

### Cluster 3

**Figure 3:**
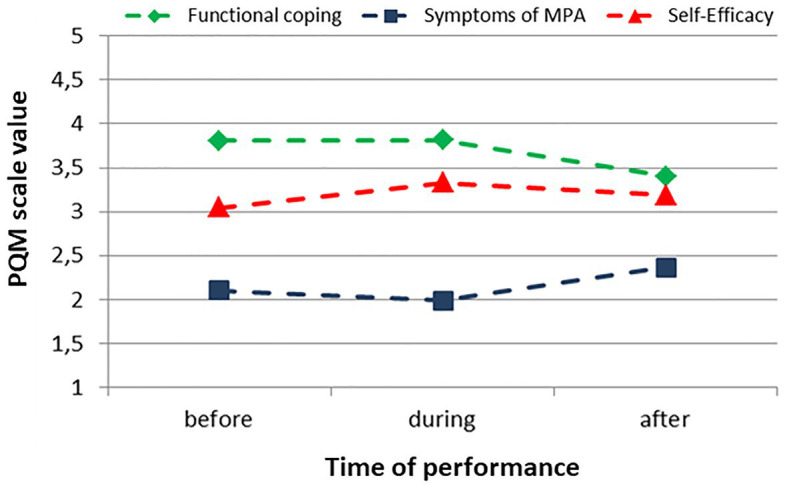
Cluster 3, progression of the values of the PQM scales before, during, and after the performance (*n* = 121).

Participants in Cluster 3 began their performance with moderate values for the symptoms of MPA and functional coping but with a rather low self-efficacy, which only increased slightly during the performance. After the performance, the values of the symptoms of MPA increased to the highest value of the performance and the values of the functional coping had the lowest values. The remaining 23.2% of the participants were assigned to Cluster 3 (Figure 3).

### Experienced Importance and Difficulty of the Performance

The mean values for experienced importance and difficulty of the performance and the self-assessments of musical quality are shown separated by cluster in Table 3.

**Table 3:** Variables of age, difficulty, and importance of the performance, and self-assessment of musical performance across the three clusters.

Variables	Cluster 1 (*n* = 259)	Cluster 2 (*n* = 142)	Cluster 3 (*n* = 121)
Age (in years)	32.8 (14.6)	28.8 (12.4)^**^	32.7 (14.8)
Personal importance	3.1 (0.7)	3.2 (0.6)	2.7 (0.7)^**^
Compared performance difficulty	2.0 (0.9)	2.4 (0.9)^**^	2.1 (1.0)
Concert difficulty	2.8 (0.6)^**^	3.1 (0.5)	3.0 (0.8)
Self-assessment of musical quality	4.7 (0.6)	4.5 (0.9)	4.1 (0.6)^**^

In brackets: SD of the mean; *n* = 522.

** *p* < 0.01.

In the individual importance of the performance, there was a significant effect found between the three clusters [*F*(2,505) = 19.945, *p* < 0.001]. For musicians in Cluster 3, the performance was significantly (Tukey HSD; *p* < 0.001) less important than for musicians in Clusters 1 and 2, although all three clusters rated the performance as being personally rather important.

The compared performance difficulty showed also a significant effect across the clusters [*F*(2,505) = 7.867, *p* < 0.001]. The musicians from Cluster 2 evaluated the performance to be more difficult than other performances than the musicians of Clusters 1 and 3 (Tukey HSD; *p* < 0.001). This corresponds to the more strongly pronounced symptoms of MPA with Cluster 2.

In the question to rate the general concert difficulty, there was again a significant effect of cluster [*F*(2,505) = 10.506, *p* < 0.001]. For the musicians in Cluster 1 the difficulty was significantly lower (Tukey HSD; *p* = 0.002) compared to the ratings of musicians in the other two clusters.

### Age and Gender

In terms of age there were significant differences between the clusters [*F*(2,506) = 6.677, *p* = 0.002]. The participants of Cluster 2 were significantly younger compared to Clusters 1 and 3 (*p* = 0.002; *p* = 0.014, respectively), whereas the mean age of Clusters 1 and 3 did not differ (Table 3).

When sorted by gender, there were no significant differences in the distribution between the clusters (*χ*^2^ = 3,168, *p* = 0.204).

### Distribution of Musical Subgroups Across the Clusters

The cluster distribution of the three musical subgroups showed highly significant differences (*χ*^2^ = 40.486, *p* < 0.001). The distribution percentages of the subgroups across the clusters are shown in Table 4. As the clusters as well as the subgroups have different sizes, the percentages of the subgroups within each cluster were compared to the overall percentage of that cluster. A positive difference indicates that more musicians of this subgroup are in that cluster than are in the total sample. A negative difference represents less musicians in the cluster than in the total sample. The differences show that most of the choir singers are in Cluster 1 (+16.8%). A larger amount of non-professional orchestra musicians are in Cluster 2 (+8.4%), and Cluster 3 contains more of the professional orchestra musicians (+11.1%) than compared with the total distribution.

**Table 4:** Distribution of the musician’s subgroups across the three clusters in percent.

	Cluster 1		Cluster 2		Cluster 3	
	% in Cluster	Diff.	% in Cluster	Diff.	% in Cluster	Diff.
Professional orchestra musicians (*n* = 137)	46.7%	−2.9%	19.0%	−8.2%	**34.3%**	**+11.1%**
Non-professional orchestra musicians (*n* = 236)	40.7%	−8.9%	**35.6%**	**+8.4%**	23.7%	+0.5%
Amateur choir singers (*n* = 149)	**66.4%**	**+16.8%**	21.5%	−5.7%	12.1%	−11.1%
Total across the clusters	49.6%		27.2%		23.2%	

Diff.: difference to the total percentage in the cluster in the last row; bold: highest difference in the row.

### Distribution of the Variable “Solo Part” and Self-Assessment of Musical Quality of the Performance Across the Clusters

A significant distribution difference of musicians with a solo part during the performance was found between the clusters (*χ*^2^ = 8.554, *p* = 0.014). Similar to the distribution of the musical subgroups, the percentages of the solo and non-solo musicians in each cluster were compared to the total cluster distribution (Table 5). The differences showed that more musicians who did not play a solo were in Cluster 1 (+3.7%). In contrast, musicians with a solo part were found more in Cluster 2 (+9.3%) than usually distributed across the total sample.

**Table 5:** Distribution of the musicians playing a solo part during the performance across the clusters.

Playing a solo part during the performance	Cluster 1		Cluster 2		Cluster 3	
	% in Cluster	Diff.	% in Cluster	Diff.	% in Cluster	Diff.
Yes (*n* = 139)	39.6%	−10.0%	**36.7%**	**+9.3%**	23.7%	+0.8%
No (*n* = 375)	**53.3%**	**+3.7%**	24.0%	−3.4%	22.7%	−0.3%
Total across the clusters	49.6%		27.4%		23.0%	

Diff.: difference to the total percentage in the cluster in the last row; bold: highest difference in the row.

There was a significant effect found for the self-assessment of musical quality between the clusters [*F*(2,492) = 31.746, *p* < 0.001; Table 3]. Musicians from Cluster 3 ranked themselves as significantly comparatively worse in the self-assessment of musical quality than participants of Clusters 1 and 2 (Tuckey HSD; *p* < 0.001). Additionally, Cluster 2 rated their musical quality significantly lower than Cluster 1 (Tukey HSD; *p* = 0.017).

The musicians with a solo part rated their musical quality of the performance in the self-assessment scale with 4.72 (*SD* = 0.89) significantly higher than the musicians without solo [*M* = 4.39; *SD* = 0.67; *F*(1,495) = 19.258, *p* < 0.001].

### Distribution of Instruments/Singing Across the Clusters

The distribution of the clusters according to instrumental groups revealed a significant difference (*χ*^2^ = 26.795, *p* = 0.001). Since the amount of musicians in the instrumental/singing groups and between the clusters was not evenly distributed, the percentages of the musicians across the clusters were presented and compared to the total sample distribution (Table 6).

**Table 6:** Distribution of the musical instruments across the clusters.

	Cluster 1		Cluster 2		Cluster 3	
	% in Cluster	Diff.	% in Cluster	Diff.	% in Cluster	Diff.
Strings (*n* = 222)	43.2%	−6.8%	27.9%	+0.2%	**28.8%**	**+6.8%**
Woodwind players (*n* = 60)	43.3%	−6.7%	**33.3%**	**+5.6%**	23.3%	+1.1%
Brass (*n* = 45)	35.6%	−14.4%	**37.8%**	**+10.0%**	26.7%	+4.4%
Percussion (*n* = 14)	**64.3%**	**+14.3%**	28.6%	+0.8%	7.1%	−15.1%
Singing (*n* = 149)	**66.4%**	**+16.4%**	21.5%	−6.3%	12.1%	−10.2%
Total across the clusters	50.0%		27.7%		22.3%	

Diff.: difference to the total percentage in the cluster in the last row; bold: highest difference in the row.

The differences showed that brass players were more in Cluster 2 (+10.0%). String players occurred more often in Cluster 3 (+6.8%). The percussionists and singers showed the highest appearance in Cluster 1 (+14.3 and +16.4%, respectively). The woodwind musicians were more distributed in cluster 2 (+5.6%).

## Discussion

**Figure 4:**
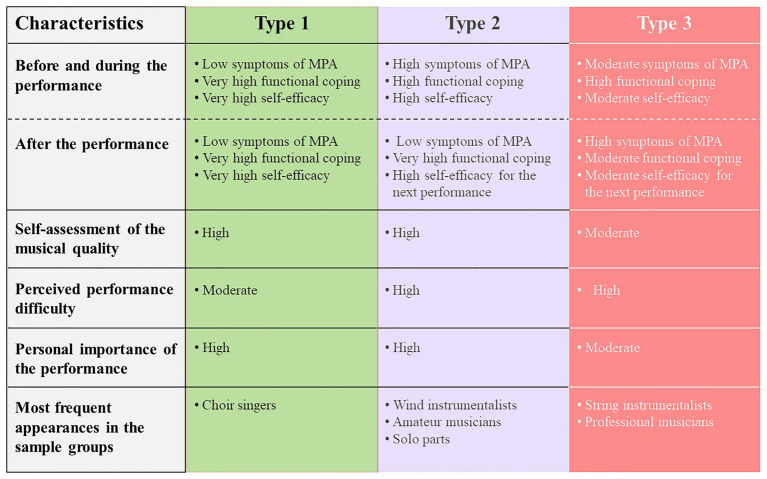
Characteristics of the three types of music performance anxiety (MPA) – summarizing the results of the cluster analysis.

The results of the cluster analysis from the pre-performance values regarding symptoms of MPA, functional coping and self-efficacy yielded three different types of MPA, which also lead to significantly different values in the evaluation after the performance. An overview of the results is shown in Figure 4.

### Description and Comparison of the Three Types of MPA

Findings of the cluster analysis indicate that there are three different types of MPA in the investigated performances:

Type 1 describes musicians who begin their performance with a positive initial constellation and follow this positively through to the end. Musicians of this type have few symptoms of MPA, can cope with MPA well, and have a stable and well-developed self-efficacy. In particular, the constellation after the performance offers good conditions for the upcoming performance. Type 1 reflects a positive performance experience that inspires the musicians for the next performance. Following the performance self-efficacy is very high, which means that these musicians feel strong for the next performance. Type 1 can be rated overall as positive.
Type 2 describes musicians who begin their performance with rather high symptoms of MPA, but can positively reduce these by use of strong self-efficacy and functional coping. Through this these musicians also achieve a positive constellation after the performance. Type 2 describes musicians in a performance that by activating resources of functional coping and positive self-efficacy was altogether a positive experience and which inspires them for the next performance. Type 2 can be rated as positive.
Type 3 describes musicians who begin their performance with moderate symptoms of MPA, but since self-efficacy and functional coping is not strong enough, they have more symptoms of MPA following the performance. Functional coping und self-efficacy is only moderately pronounced and therefore these musicians find themselves after the performance in an adverse constellation for the next performance. Type 3 describes musicians in a performance, which, although it began with only moderate MPA, could not be followed through in a positive way. Type 3 is therefore rated as critical.

#### Self-Efficacy and Functional Coping

Due to the positive effects of self-efficacy on MPA and performance success that have been demonstrated in previous studies (McCormick and McPherson, 2003; McPherson and McCormick, 2006; Hewitt, 2015; Miksza, 2015; Bugos et al., 2016), it can also be assumed in the study presented that the extent of self-efficacy is decisive for the course of MPA and that it significantly influences the resulting type. This is particularly evident in Type 2, in which the symptoms of MPA during and after performance were probably able to be reduced by high self-efficacy.

When evaluating the types, we interpret in particular the constellation after the performance as significant. Especially, functional coping and positive self-efficacy following the performance have a critical influence on how a musician feels going into the next performance. This positive attitude may lead to the opinion of seeing the performance as a challenge, which in turn increases self-confidence and has a facilitating effect on MPA (Osborne and McPherson, 2018).

Functional coping with MPA, which was differently pronounced between the three types, means cognitive strategies like strengthening positive thoughts and stopping negative thoughts on the performance, to control agitation, to stay calm, and to concentrate on communication with the audience. Functional coping had a positive impact on the course of MPA in Types 1 and 2.

### Musical Self-Assessment, Playing Solo Parts and Attitudes Toward the Performance Across the Three Types of MPA

#### Self-Assessment of Musical Quality of the Performance

The musical quality of the performance across all three types was assessed as “good” to “very good.” However, the self-assessments differ significantly among the three types. The musicians from Type 3 judged their musical performance comparatively as the lowest, while musicians in Type 1 judged theirs the best. The musicians in Type 2 lay in between. In contrast to the results of Kaleńska-Rodzaj (2018), in our data, the extent of the symptoms of MPA prior to the performance was not decisive for the assessment of the musical quality, but rather the constellation after the performance.

Self-assessment of the musical quality seems to be strongly linked to past self-efficacy, because research suggests that enactive mastery experience is a most influential source of self-efficacy (Bandura, 1997; Hendricks, 2014). Seen like this, musicians of Type 3 show a close interaction between negative preconditions for a successful performance.

#### Playing Solo Parts During the Performance

Playing a solo part was associated with more symptoms of MPA prior to the performance. Therefore, there were significantly more musicians from Type 2 who played a solo, and conversely significantly fewer musicians from Type 1 who had to play a solo. It is well known that solo performances lead to an increase in symptoms of MPA. Previous research showed that the expression of MPA varies by performance setting, with the most anxiety reported during solo performances (Cox and Kenardy, 1993; Nicholson et al., 2015).

#### Attitudes Toward the Performance

Musicians of all three types indicated that in comparison to other performances, this performance was not so easy. However, Type 2 musicians reported a significantly higher value, i.e., they experienced the requirements of the performance as most difficult. This goes along with higher symptoms of MPA prior to the performance in Type 2.

In reference to the second question about the difficulty of the performance as measured by the personal levels of performance, musicians from Type 1 found the performance significantly less challenging than musicians from the other two types. It is therefore understandable that these musicians have the fewest symptoms of MPA compared to the other two types.

Musicians from Type 3 reported that the performance was less important to them compared to musicians from Type 1 and Type 2. This indicates that the amount of symptoms of MPA seems to be less related to the importance of the performance. However, as this type also contains more professional musicians, it would be possible that the importance in less relevant due to the given prescribed performances.

### Musicians’ Subgroups, Age, and Gender Across the Three Types of MPA

#### Professional Orchestra Musicians, Non-professional Orchestra Musicians, and Choir Singers

The greatest portion of Type 1 musicians consists of choir singers, Type 2 of non-professional orchestra musicians, and Type 3 of professional orchestra musicians. Corresponding to the age distribution into these subgroups and because the sample of non-professional orchestra musicians mainly consisted of students, the musicians from Type 2 were significantly younger than those in Types 1 and 3. In none of the three groups were there more women or men.

#### Musical Instruments

Among the different groups of musical instruments, remarkably more string players landed in Type 3, more brass players in Type 2, and more percussionists in Type 1. Woodwind players were distributed almost evenly among all three types.

When assigning the types, the data indicates that the string players, who have the highest count among the professional orchestra musicians in Type 3, could represent most of the characteristics of this type. This type also consists of older musicians, who are exposed to high demands at work. This type showed the highest symptoms of MPA.

Wind players, on the other hand, are apparently more able to positively handle occurring symptoms of MPA, as they are predominantly in Type 2.

The choir singers form their own group. They differ from the other subgroups by a high representation in Type 1. Since these are non-professional choirs, it is conceivable that the shared experience in the choir community leads to positive effects. Thus, symptoms of MPA have also been described for choir singers (Kenny et al., 2004; Ryan and Andrews, 2009). A study with 201 semi-professional choir singers comparable to the sample in our study showed that MPA was a common experience for these choral singers. Solo performances were reported to be more anxiety inducing than ensemble experiences, but performing in instrumental ensembles induced greater MPA than in choral ensembles (Ryan and Andrews, 2009).

### Limitations of the Study

When interpreting the results, it should be noted that the assigned type refers to *one* performance, after which the musicians filled out the questionnaire. There is still missing research regarding repeated measuring of the same musicians across multiple performances. However, the major focus of this preliminary study was on the large cluster analysis and the *post hoc* analyses on the cluster distributions to identify certain aspects of types of MPA across a large number of musicians.

There are also limitations in the addressed musical subgroups. The study mainly had a tendency of more classically oriented orchestra musicians. The addition of other musicians, including musical instruments such as the piano and the guitar, would be a necessary improvement.

## Conclusion

Findings indicate that there are three different types of MPA. It was shown that self-efficacy seems to play an important role in keeping symptoms of MPA low. The results indicate that self-efficacy acts as some kind of moderator variable: high symptoms of MPA before the performance could be reduced by a strong self-efficacy in the course of the performance. This finding, however, should be investigated more in further research. In particular, it could be of interest to investigate the effect of interventions to improve self-efficacy on MPA. Furthermore, the results provide new starting points for a differentiated understanding of MPA. They provide certain insights on how musicians perceive aspects of MPA at performances. In subsequent studies, it would be recommended to examine to what extent the type assignment of the musicians remains stable over several performances.

## Data Availability Statement

The raw data supporting the conclusions of this article will be made available by the authors, without undue reservation, to any qualified researcher.

## Ethics Statement

The studies involving human participants were reviewed and approved by Ethics Committee of the University Clinic Freiburg. Written informed consent for participation was not required for this study in accordance with the national legislation and the institutional requirements.

## Author Contributions

FK did mainly the data collection. CS and MN performed the statistical analyses. All authors contributed to the article and approved the submitted version.

### Conflict of Interest

The authors declare that the research was conducted in the absence of any commercial or financial relationships that could be construed as a potential conflict of interest.
